# Natural variants of von Willebrand factor R1205 causing von Willebrand disease with accelerated von Willebrand factor clearance: *In silico* docking models and energetics of the interaction with both LRP1 and GpIb A1 domain

**DOI:** 10.1371/journal.pcbi.1013458

**Published:** 2025-12-03

**Authors:** Monica Sacco, Stefano Lancellotti, Antonietta Ferretti, Maria Basso, Leonardo Di Gennaro, Giancarlo Castaman, Raimondo De Cristofaro

**Affiliations:** 1 Dipartimento di Medicina e Chirurgia Traslazionale, Università Cattolica S. Cuore, Facoltà di Medicina e Chirurgia “Agostino Gemelli”, Roma, Italy; 2 Servizio Malattie Emorragiche e Trombotiche, Fondazione Policlinico Universitario “A. Gemell” IRCCS, Roma, Italy; 3 Centro Malattie Emorragiche e della Coagulazione, Dipartimento di Oncologia, Ospedale Universitario “Careggi”, Firenze, Italy; Indonesia International Institute for Life Sciences, INDONESIA

## Abstract

Type 1 von Willebrand disease (VWD) is often caused by variants in von Willebrand factor (VWF), including p.R1205H (“Vicenza mutation”), which accelerate VWF clearance via macrophage receptor LRP1 and impair platelet adhesion and activation. However, the structural mechanisms underlying these phenotypes remain partially unclear. Here, we use integrative computational modeling (I-TASSER, HADDOCK2.4, and PRODIGY) to predict how p.R1205H/C/L/S variants alter VWF interaction with LRP1 and its platelet receptor GPIbα that binds to the A1 VWF domain. Our models reveal that R1205 acts as a structural hinge: its variants disrupt polar networks in VWF’s D3 domain, exposing neo-epitopes that enhance LRP1 binding (ΔΔG up to −5.3 kcal/mol) while destabilizing the A1 domain’s α1-β2 loop, reducing GPIbα affinity (≅30-fold for R1205L/C). These findings explain clinical observations, p.R1205H rapid clearance yet retained platelet adhesion, and establish R1205 as a dual-functional switch regulating VWF circulatory lifetime and hemostatic activity. This analytical procedure provides a template for predicting pathogenicity of VWF variants and designing targeted therapies for VWD.

## Author summary

In our study, we explored how specific genetic changes in von Willebrand factor (VWF), a protein crucial for haemostsis, lead to a bleeding disorder called von Willebrand disease (VWD). We focused on amino acid changes at position R1205 in VWF, known to cause rapid clearance of the protein from the bloodstream, resulting in low levels and impaired haemostasis. Using advanced computer modeling, we investigated how these variants alter VWF’s interactions with two key partners: LRP1, a receptor that removes VWF from circulation, and GpIbα, a platelet receptor essential for platelet adhesion and activation. Our models revealed that R1205 acts like a structural hinge. Mutations disrupt its stabilizing role, exposing regions that bind more tightly to LRP1, thus explaining the accelerated clearance seen in patients. Simultaneously, these changes can also weaken VWF’s ability to interact with platelets, further impairing platelet haemostatic function. This work provides a blueprint for understanding how some VWF variants cause disease and could guide the design of targeted therapies to correct or compensate for these defects, improving treatment for VWD patients.

## Introduction

The high molecular weight glycoprotein VWF is essential to maintain normal hemostasis through its ability to mediate platelet-subendothelial interactions as well as interaction with platelets to support their aggregation at the site of damaged endothelium. Type 1 von Willebrand disease (VWD1) is characterized by a partial quantitative deficiency of VWF, with plasma levels typically ranging from 5% to 50% of normal [1]. This phenotype arises from diverse pathogenic mechanisms, including impaired synthesis, intracellular degradation, defective secretion, accelerated plasma clearance, or null alleles [2]. “Vicenza” VWD is a well-characterized subtype of VWD1, caused by the p.R1205H missense variant in the D3 domain of VWF. This variant is associated with markedly accelerated clearance of VWF from the circulation, leading to disproportionately low plasma VWF antigen (VWF:Ag) and ristocetin cofactor activity (VWF:RCo), often ≤0.10 U/mL, and reduced Factor VIII levels (<0.30 U/mL) [3–7].

Despite these low plasma levels, platelet VWF content and function remain normal, contributing to a variable bleeding phenotype [1,8,9]. Other types 1 VWD variants occurring at the same place, such as p.R1205C, p.R1205L, and p.R1205S share similar characteristics with VWD Vicenza, being also characterized by comparable clearance-accelerated characteristics, supporting the critical role of R1205 in regulating VWF half-life [3,10]. This pathogenetic mechanism was demonstrated via desmopressin (DDAVP) studies [2] and human recombinant protein infusion in the VWF knockout mouse [11], as well as through high VWFpp/VWF:Ag ratios, observed in patients’ plasma [4,12]. The p.R1205H missense variant occurs in the E3 module of the VWF D3 domain [2]. This pathological variant in some cases shows even alterations in the typical multimer pattern in SDS-agarose gels with a predominance of ultra-large VWF multimers [4,13], Also these changes were attributed to the very rapid clearance of VWF multimers [7,12,14] that limit their physiological proteolytic processing by ADAMTS-13 [15,16]. The direct role of the arginine residue at position 1205 of the mature VWF molecule in regulating the half-life of the protein was confirmed in several studies using VWF -/- mice. The mean half-life of human p.R1205H VWF was markedly shorter than that of human WT-VWF in VWF -/- mice [11]. The structural basis for these clinical observations remains incompletely understood. The R1205 residue is located within the D3 domain, which in previous studies was shown to exert a conformational restraint on the A1 domain and modulate its affinity for GPIbα [25–27]. Amino acid change at R1205 may disrupt this intramolecular regulation, leading to enhanced binding to clearance receptors, such as LRP1, while impairing platelet adhesion.

Based on the above findings, this study employs integrative computational modeling to investigate how p.R1205H/C/L/S variants alter VWF interactions with both LRP1 and GPIbα.

## Results

### Molecular modeling of WT and R1205 pathological variants of VWF

**Fig 1:**
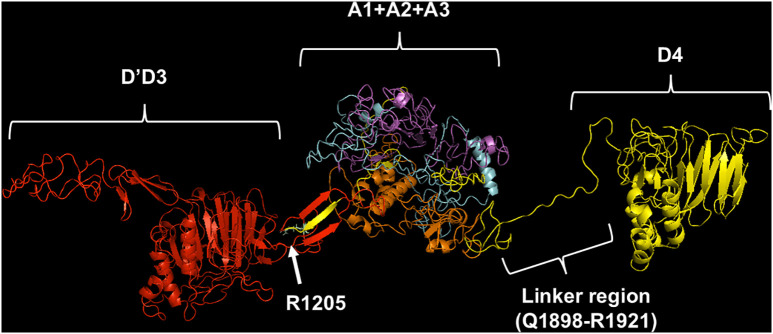
Best model of the 764-2191 region of WT-VWF. The three globular regions involving a) the D’D3, b) the entangled A1-A2, and A3 domains, and c) the connecting region with the D4 domain are shown. The D’D3 domain is shown in red, the A1 in magenta, the A2 in cyan, the A3 in orange, where some regions, involving the D4 domain, whose structure was not yet solved, are shown in yellow. The side chain of R1205 is shown in palegreen. The model was obtained with the I-TASSER program, whereas the manipulation was accomplished with PyMOL.

Table 1 lists the TM score and RMSD values for both the WT and VWF variants. All 3D models had a template modeling score (TM-score) >0.50, indicating acceptable structural topology. Only the p.R1205C variant exhibited a TM-score at the limit of structural acceptability. Overall, the topology of the VWF forms is roughly characterized by three globular parts: 1) domain D’D3 (764–1242, pdb #6N29); 2) the entangled region comprising the A1 domain (1263–1468, pdb #1AUQ), the A2 domain (1469–1685 pdb#3GXB), and the A3 domain (1686–1874 (pdb #1ATZ); 3) the D4 domain (1875–2191, whose molecular structure was not yet experimentally solved (Fig 1).

**Table 1:** Results of the I-TASSER analysis concerning the 3D models of the Monomer WT (764-2191) and p.R1205X VWF variants.

Species	Estimated TM-score	Estimated RMSD (Å)	S764-V2191 distance (Å)
**WT**	0.53 ± 0.14	10.1 ± 4.6	189
**p.R1205H**	0.52 ± 0.11	10.5 ± 4.8	194.5
**p.R1205S**	0.56 ± 0.15	15.4 ± 3.4	168
**p.R1205C**	0.45 ± 0.15	15.5 ± 3.3	194.9
**p.R1205L**	0.81 ± 0.09	8.0 ± 4.4	108.6

**Fig 2:**
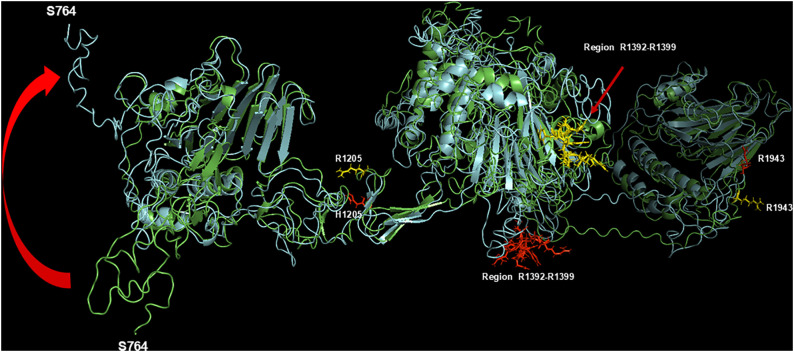
Superposition of WT-VWF (green ribbon) and p.R1205H (cyan ribbon). The region R1392-R1399 and R1943, involved in binding to LRP1 domain IV, are shown as yellow sticks and red sticks for WT and p.R1205H VWF, respectively. The R1943 side chains of both WT (yellow sticks) and p.R1205H variant (red sticks) are also shown. Note the large topological change for the region S764-T789, likely deriving from a huge conformational flexibility (curve red arrow). The superposition and alignment of the two structures were performed using the PyMOL program.

These globular parts are connected by two linker regions (1181–1210 and 1898–1921), as shown in Fig 1. As to the R1205X variants, analysis of the molecular modeling results showed that the substitution of the Arg with different amino acids alters the conformation of discrete regions of the molecule, as, for instance, in the case of p.R1205H (see Fig 2). However, the TM-align program provided in all cases a TM-score value ranging from 0.64 (p.R1205L) and 0.84 (p.R1205C), with intermediate values for p.R1205H (0.76) and p.R1205S (0.75). These values show that all these VWF mutants have roughly the same overall structure [17].

**Fig 3:**
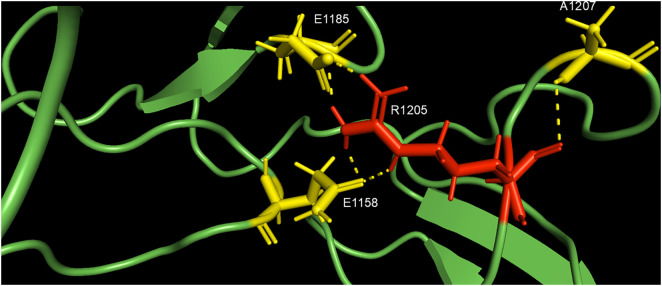
Magnification of the WT-VWF segment encompassing R1205, from the model shown in Fig 1. Polar interactions ≤3 Å (dashed lines) between the side chain of R1205 (shown in red sticks) and the surrounding amino acids (yellow sticks) are shown. The model was obtained with I-TASSER and manipulated with PyMOL.

**Fig 4:**
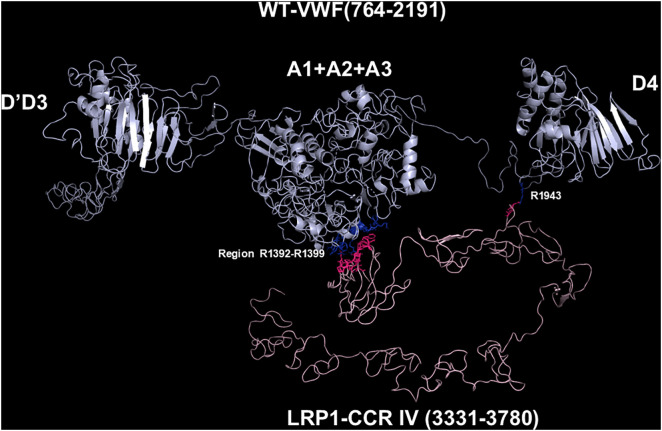
Molecular models obtained with I-TASSER and Haddock 2.4 programs of the adduct between WT-VWF and CCR IV of LRP1. The VWF construct is shown in cyan, whereas the CCR IV of LRP1 is shown in pink. The interacting residues of the complex, all at distances comprised between 1.57 and 3.78 Å, are listed in S2 Table.

In the WT-VWF molecule, R1205 forms a series of polar and salt bridges at a distance ≤3 Å with A1207, E1185 and E1158 (see Fig 3). This setting contributes to stabilizing a correct topology of the region between the D’D3 domain and the first linker region (aa 1181–1210). The lack or change of these bonds in the other R1205X variants allosterically induces the rearrangement of regions involving both the segment R1392-R1399 and R1943, involved in binding to LRP1 cluster IV (see Fig 4). The relevance of the positive charge at VWF-R1205 has been recently remarked by the results obtained with SPR experiments on WT VWF-LRP1 interaction by Atiq et al. [7]. Hence, the conformational effects arising from substitution of R1205 with different amino acids would determine the functional changes in the interaction with LRP1 (see below). The changed polar interactions of the side chains of H1205, C1205, L1205, and S1205 with surrounding residues are shown in S1–S4 Figs. In these cases, the lack of interactions of the side chain of R1205 with surrounding amino acids causes long-range conformational changes, as shown in S5 Fig.

The same computational methodology described above was used to predict the *ab initio* structure of LRP1 receptor cluster IV (V331–P3780).The best final model is shown in the S6 Fig.

The PDB files for all structural models are available for download from the PDBsum Generate server (https://www.ebi.ac.uk/thornton-srv/databases/pdbsum/Generate.html), using the “pdb code” and password listed for each structure in S1A Table of the Supporting Information file.

### Models of the complexes between LRP1 Cluster IV and the VWF species

**Fig 5:**
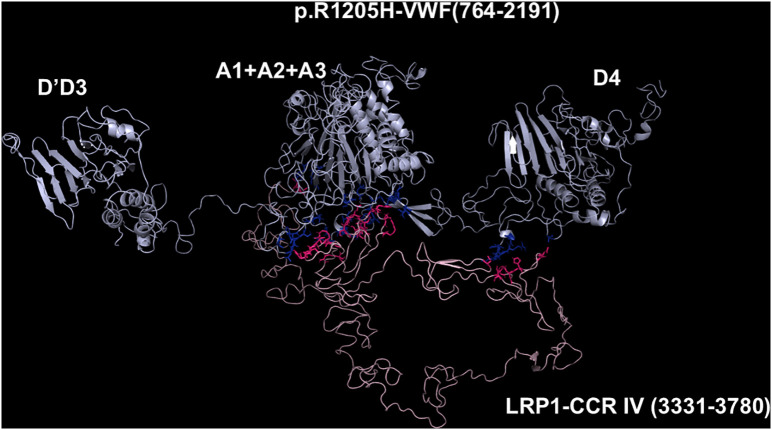
Molecular models obtained with I-TASSER and Haddock 2.4 programs of the adduct between p.R1205H-VWF and CCR IV of LRP1. The VWF construct is shown in cyan, whereas the CCR IV of LRP1 is shown in pink. The interacting residues of the complex, all at distances comprised between 1.60 and 3.45 Å, are listed in S2 Table.

**Fig 6:**
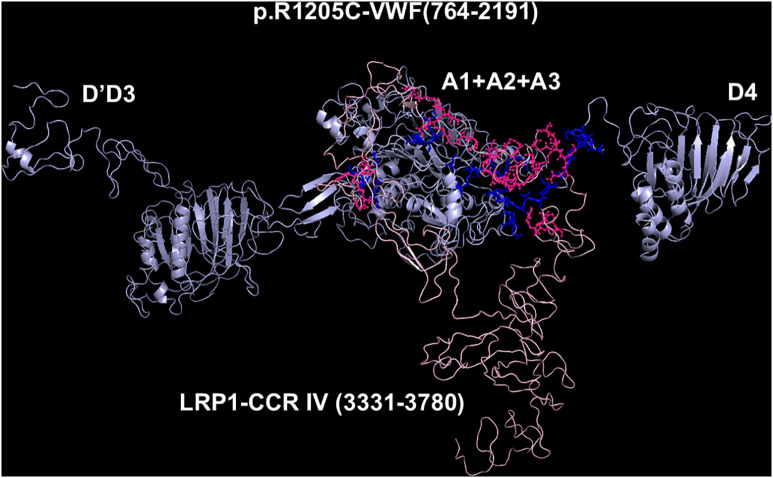
Molecular models obtained with I-TASSER and Haddock 2.4 programs of the adduct between p.R1205C-VWF and CCR IV of LRP1. The VWF construct is shown in cyan, whereas the CCR IV of LRP1 is shown in pink. The interacting residues of the complex, all at distances comprised between 1.58 and 3.73 Å, are listed in S2 Table.

**Fig 7:**
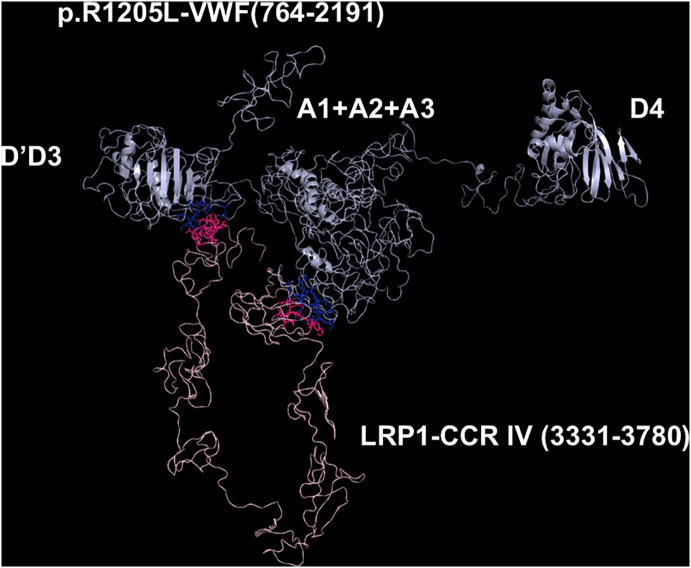
Molecular models obtained with I-TASSER and Haddock 2.4 programs of the adduct between p.R1205LVWF and CCR IV of LRP1. The VWF construct is shown in cyan, whereas the CCR IV of LRP1 is shown in pink. The interacting residues of the complex, all at distances comprised between 1.56 and 3.87 Å, are listed in S2 Table.

**Fig 8:**
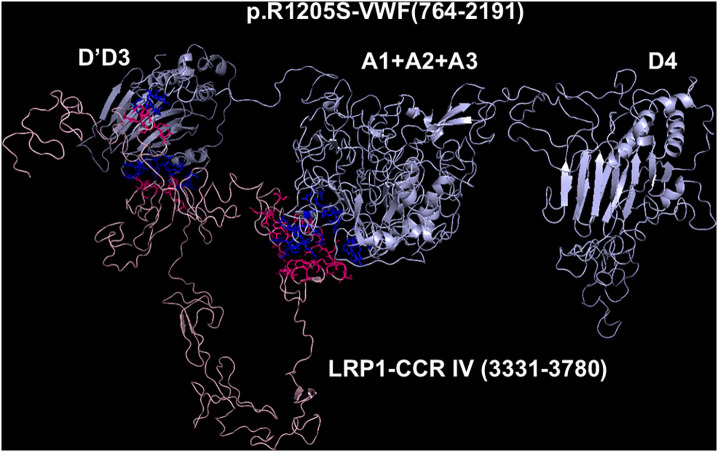
Molecular models obtained with I-TASSER and Haddock 2.4 programs of the adduct between p.R1205SVWF and CCR IV of LRP1. The VWF construct is shown in cyan, whereas the CCR IV of LRP1 is shown in pink. The interacting residues of the complex, all at distances comprised between 1.59 and 2.61 Å, are listed in S2 Table.

**Fig 9:**
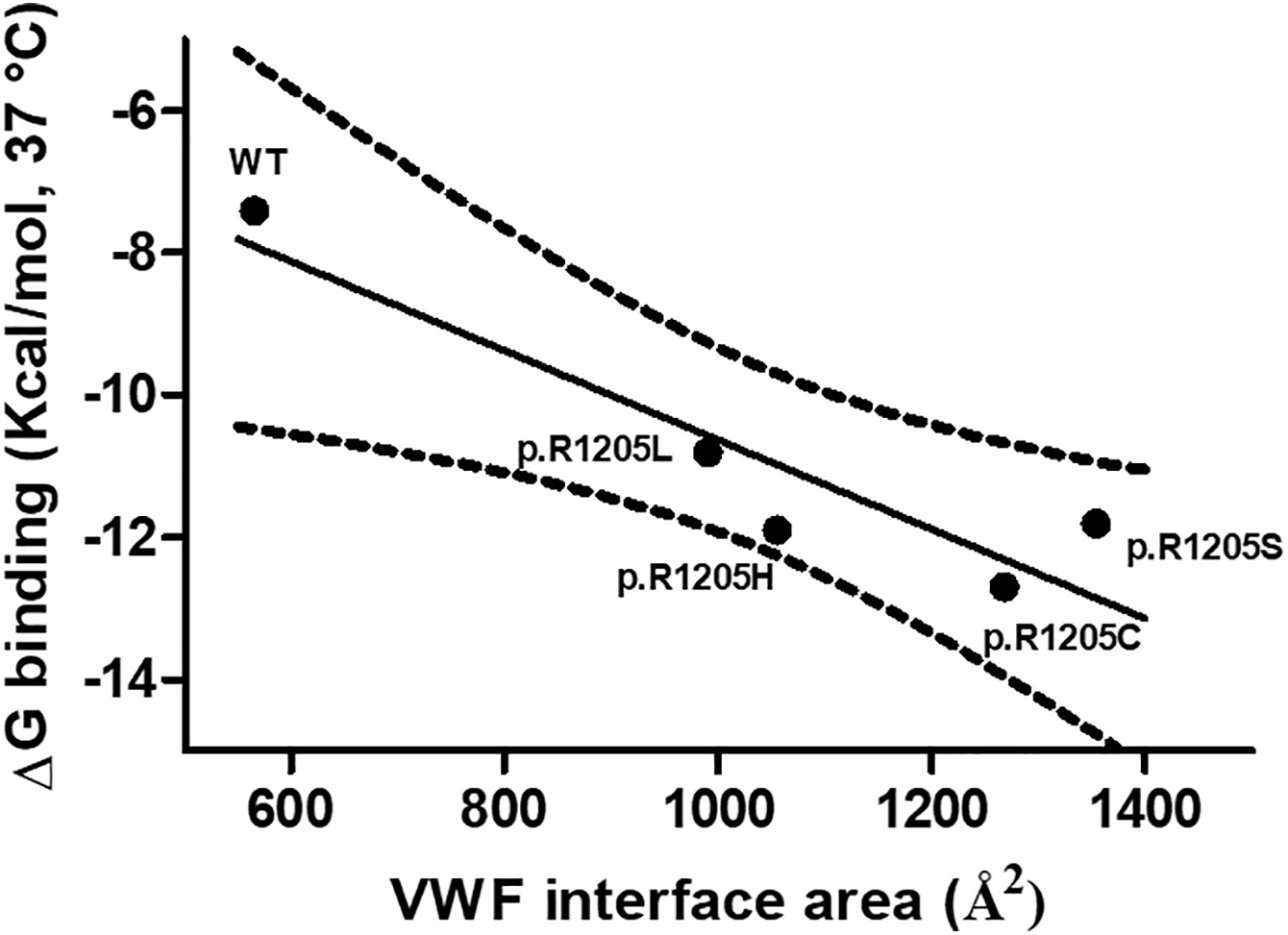
Values of ΔG of binding of the VWF forms to LRP1 as a function of VWF interface areas. The slope of the linear regression is -0.006271 ± 0.001458 (p = 0.0231). The dashed lines represent the 95% confidence interval of the regression.

As paradigmatic adducts between LRP1 cluster IV and the VWF models, we selected those characterized by highest negative HADDOCK and Z-scores. Figs 4–8 shows the best models obtained using the HADDOCK platform. In both WT-VWF and p.R1205H variants, the entangled A1-A2-A3 and D4 domains are engaged in interaction with LRP1, whereas the p.R1205L and p.R1205S engage the D’D3 and the entangled A1-A2-A3 domains (Figs 4, 5, 7 and 8). At variance, the p.R1205C mutant interacts with LRP1 cluster IV with the A1-A2-A3 domain only (Fig 6). The engagement of different regions of VWF for the interaction with LRP1 inevitably changes the energetics of binding. S2 Table lists all the hydrogen bonds and salt bridges present in all the complexes formed by all the VWF forms and LRP1. WT-VWF interacts with the sequence 1392–1399, along with R1943. All VWF mutants except p.R1205C also engage R1399 for binding to LRP1, though they utilize additional regions due to conformational changes induced by the mutations (see S2 Table for details). Table 2 shows that the VWF interaction area for LRP1 binding is significantly larger for the R1205 variants than for the WT form, changing from about 570 Å^2^ up to 1350 Å^2^ in p.R1205S mutant. Because of the conformational effects, there is a strong increase of the predicted interaction affinity of the VWF variants compared to the WT form. The binding affinity of different VWF forms exhibits a linear dependence on the interface area, as shown in Fig 9. The ΔG of equilibrium binding constants of both WT- and p.R1205X variants are reported in Table 3 and shown in S7 Fig. It is important to note that the PRODIGY algorithm, used to calculate binding free energies (ΔG), has a reported root-mean-square error (RMSE) of approximately 1.2 - 2.0 kcal/mol when benchmarked against experimental data [18]. This level of uncertainty, inherent to computational affinity predictions, translates to an approximate order-of-magnitude variability in the calculated dissociation constants (Kd). While this uncertainty affects the precise quantitative estimates, the large, predicted differences in ΔG between wild-type and variant VWF (e.g., a ΔΔG of ~4.5 kcal/mol for p.R1205H) are substantially greater than the typical error margin, providing confidence in the central finding that the R1205 variants significantly enhance binding affinity for LRP1. The PDB files for all structural models are available for download from the PDBsum server using the username and password provided in S1 Table of the Supporting Information. The full statistics of the molecular models obtained using the HADDOCK program are reported in S3 Table in Supporting Information file.

**Table 2:** Interface features of the adducts between WT and VWF variants at R1205 and LRP1 domain IV. Chain A represents VWF, whereas Chain B is LRP1 domain IV.

VWF species	Chain	No. of Interface residues	Interface Area (Å)	No. of Salt bridges	No. of hydrogen bonds	No. of Non-bonded contacts
	**A**	9	565	7	12	78
**WT**	**B**	11	590			
	**A**	23	901	6	14	127
**p.R1205H**	**B**	20	1015			
	**A**	21	1268	2	6	82
**p.R1205C**	**B**	23	1265			
	**A**	14	990	6	12	102
**p.R1205L**	**B**	21	907			
	**A**	21	1354	1	8	77
**p.R1205S**	**B**	23	1345			

**Table 3:** Calculated ΔG of binding of different WT monomer species and LRP1-Cluster IV with the corresponding K_d_ values calculated by the PRODIGY program (T = 37 °C).

Species	ΔG (Kcal mol^-1^)	K_d_ (M)
WT	-7.4	5.8x10^-6^
p.R1205H	-11.9	4.0x10^-9^
p.R1205S	-11.8	4.9x10^-9^
p.R1205C	-12.7	1.2x10^-9^
p.R1205L	-10.8	2.4x10^-8^

### Models of the complexes between N-terminal domain of platelet GpIbα and the various VWF species

**Fig 10:**
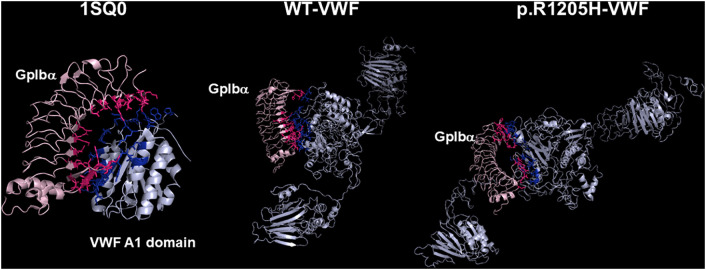
Best models of the 764-2191 region of WT-VWF and R1205H-VWF models bound to the N-terminal (1-266) domain of platelet GpIbα. For comparison, the crystal structure of the N-terminal (1-266) region of GpIbα bound to the VWF A1 domain (pdb: 1SQ0) is also shown. The side chain of interacting residues of GpIbα is shown in warm pink, whereas those of VWF are shown as blue sticks. The models were generated by the HADDOCK program, whereas the rendering was accomplished with Pymol.

**Fig 11:**
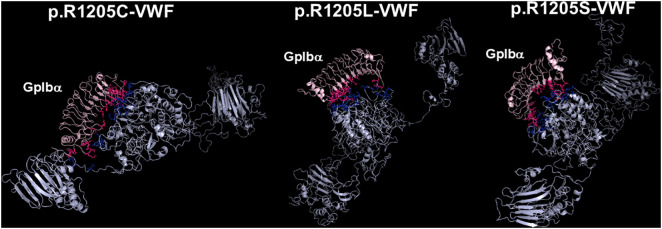
The models of the complexes of the other p.R1205X variants (p.R1205C, p.R1205L, and p.R1205S) with the GpIbα domain. In all cases, GpIbα binds to the A1-A3 region of VWF. The models of VWF(764-2191) are shown cyan, while the GpIbα molecule is shown in pink. The side chain of interacting residues of GpIbα is shown in warm pink, whereas those of VWF are shown as blue sticks. The models were generated by the HADDOCK program, whereas the rendering was accomplished with Pymol.

**Fig 12:**
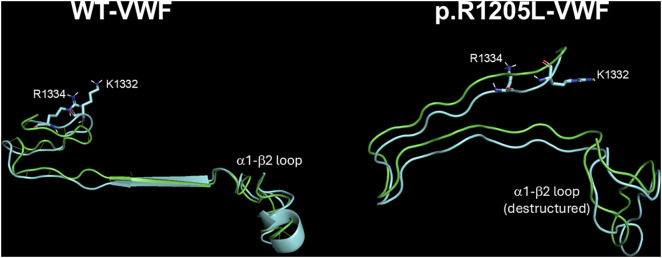
Conformational dynamics of the VWF A1 domain α1-β2 loop upon GPIbα binding. The region encompassing residues 1294–1336 is shown for both WT-VWF and p.R1205L variants (green in the unbound conformation and cyan in the GPIbα-bound states). In WT-VWF, binding to GPIbα induces a defined conformational rearrangement in the α1-β2 loop (residues 1309-1314), positioning key residues like K1332 and R1334 to form direct contacts with GPIbα. In contrast, the p.R1205L mutation destabilizes the α1-β2 loop, preventing it from adopting the stable, binding-competent conformation observed in the WT. This structural disruption, evident from the altered orientation of K1332 and R1334, explains the weakened affinity for the platelet receptor. Similar loop destabilization was observed for the p.R1205C variant. Images were rendered using PyMOL.

The deposited pdb file 1SQ0 was considered as a template for the analysis of the models of the complexes between the N-terminal portion of GpIbα (H1-T266) and the VWF(764–2191) forms. In the pdb file 1SQ0 concerning the interaction between N-Terminal GpIbα and the A1 domain of VWF several hydrogen bonds are formed between S1325, A1327, R1334, E1359, K1362, F1366, Q1367, and R1395 of VWF and M239, K237, D18, S39, Y238, P198, N226, Y228, K152, T175, and E225 of GpIbα, respectively. Thus, an extensive region of the A1-A2-A3 domain of the VWF(764–2191) molecule is involved in this interaction. Overall, this setting is also followed by the R1205X variants (see Figs 10 and 11). All p.R1205X variants exhibit reduced binding affinity for GpIbα. Compared to WT-VWF, p.R1205H displayed approximately one-third of the binding affinity. The p.R1205L, p.R1205C, and p.R1205S variants showed even lower affinities, with approximately 39-fold, 34-fold, and 11-fold reductions, respectively (Table 4). A detailed inspection of the models of both p.R1205L and p.R1205C variants shows that the conformation of the α1-β2 loop of the VWF A1 domain (E1294-D1323, G2 in the model shown in Fig 1), which plays a major role in modulating the affinity of VWF for GpIb, is largely altered in comparison to the WT form, dampening the affinity for the platelet receptor, as shown in Fig 12.

**Table 4:** Calculated ΔG of binding and the corresponding K_d_ values for the different VWF species and the N-terminal (H1-T266) domain of platelet GpIbα. The binding parameters were calculated by the PRODIGY program at T = 37 °C.

Species	ΔG (Kcal mol^-1^)	K_d_ (M)
WT	-11.7	5.6 x 10^–9^
p.R1205H	-11.1	1.5 x 10^–8^
p.R1205S	-10.2	6.0 x 10^–8^
p.R1205C	-9.5	1.9 x 10^–7^
p.R1205L	-9.4	2.2 x 10^–7^
1SQ0*	-10.1	7.2 x 10^–8^

*PDB file of the complex formed by A1 VWF domain and N-Terminal part of GpIbα.

The PDB files for all structural models are available for download from the PDBsum Generate server using the “pdb code” and password provided in S1 Table of the Supporting Information.

## Discussion

The study addresses a clinically significant question, namely how variants VWF at R1205 affect its interaction with both LRP1 and GPIbα, leading to VWD1, functionally characterized by normal or slightly prolonged bleeding time in vivo and strongly altered Platelet Function Analyzer (PFA) test in vitro [2,4,19–28]. Macrophage LRP1 mostly modulates the clearance of VWF and its complex with Factor VIII (FVIII). LRP1 would accomplish this function via direct shear stress-induced interactions with VWF while blood containing VWF is flowing along LRP1-expressing macrophages, or as part of a functional LRP1–β_2_-integrin complex, as previously demonstrated [29]. However, although this represents the predominant clearing pathway of VWF, it is not the only mechanism involved in the VWF clearance from circulation. Other systems, in fact, contribute to this function such as the asialoglycoprotein receptor, CLEC4M, SRA-1, or members of the Siglec family [14].

The present findings agree with the clinical phenotype of the “Vicenza” type 1C VWD, characterized by very rapid clearance of the p.R1205H variant. Previous studies demonstrated that in VWF-null mice, recombinant VWF with the p.R1205H mutation has a ≈ 9-fold shorter plasma residence time (0.3 h) than wild-type VWF (2.8 h). Of interest, in that study the clearance was independent of multimeric size [10]. This aligns with our finding that the p.R1205H monomer model shows much higher LRP1 affinity than WT, as also recently found in binding experiments with surface plasmon resonance [7]. Liver and spleen macrophages likely regulate VWF clearance by binding and endocytosing VWF multimers, as supported by experimental data [10,29]. Hence, this very high affinity for macrophage LRP1 would mainly contribute to playing a prominent role in clearing the VWF–FVIII complex [10]. The molecular details of the VWF regions involved in the clearance mechanism have not yet been fully defined. However, the knowledge of the functional behavior of the natural mutants associated with enhanced VWF clearance unequivocally demonstrated that the involved amino acid residues are clustered within the D’D3 and A1A2A3 domains [30]. Experimental studies demonstrated in fact that recombinant A1A2A3 domains infused in mice were cleared from circulation at a similar rate to full-length VWF [13]. Notably, the fragment D’D3A1A2A3 was cleared more slowly, suggesting that the D’D3 domains may have a regulatory role in VWF clearance, slowing the process of interaction with VWF scavenger receptors [13]. A similar role of the D’D3 domain was demonstrated for the A1-VWF domain interaction with the platelet receptor GpIb-IX-V [31]. Any variations that can perturb the native conformation of the D3 domain around R1205 can abrogate the inhibitory effect of the D’D3 domain. The results obtained in the present molecular modeling investigation concerning p.R1205H, p.R1205S, p.R1205L, and p.R1205C fully agree with this scenario. A critical question is how these variants enhance VWF affinity for macrophage LRP1. In WT-VWF, R1205 establishes both polar contacts (≤3 Å) and salt bridges with A1207, E1185, and E1158 (see Fig 3). Functioning as structural hinge, these interactions maintain proper spacing between VWF’s the D’D3, A1-A2-A3, and D4 domains (see Fig 1), while stabilizing its overall topology. Strikingly, the S764-V2191 distance shows variant-dependent huge changes: 108.6 Å (p.R1205L), 194.9 Å (p.R1205C), versus 189 Å (WT), as listed in Table 1. R1205 variant-induced conformational changes expose neo-epitopes, ultimately modifying VWF binding energetics to LRP1 domain IV. It is important to note that our current models provide a static, energetic snapshot of these interactions. Future studies employing molecular dynamics simulations and experimental binding experiments will be crucial to assess the stability and conformational dynamics of these complexes over time, further elucidating the kinetic aspects of VWF clearance. It is likely that phylogenetic pressure has selected an arginine residue at position 1205 to counteract excessive binding to macrophage receptors responsible for VWF clearance, thereby prolonging its persistence in the bloodstream.

As to the interaction with the platelet receptor GpIbα, the R1205X variants exhibit a significant decrease of their affinity for the receptor. Relative to WT-VWF, we observed 33-fold less affinity for p.R1205L/C variants, with more moderate reductions for p.R1205H (3-fold) and p.R1205S (10-fold). The p.R1205H variant is distinctive for retaining the highest residual GPIb affinity among VWF R1205X variants. Originally classified as “platelet-normal VWD1” [32], Vicenza-type VWD exhibits a unique phenotype: severe plasma VWF deficiency with normal VWF platelet stores, preserved large multimers [33] but defective RIPA, hallmarks that prompted its reclassification as type 2M. However, the clinical features of VWF “Vicenza” are still debated, because other studies found a normal or just slightly reduced functionality of the p.R1205H variant for platelets [33]. Regarding the interaction of p.R1205C/L with platelet GPIb, the EAHAD Coagulation Factor Variant Database [34] lacks specific data on potential impaired binding to GPIb and currently classifies these variants merely as “likely pathogenic”. Similarly, beyond its accelerated clearance from circulation, no data are available regarding the p.R1205S interaction with GPIb [3]. From a structural viewpoint, the α1-β2 loop in the VWF A1 domain (residues L1309-V1314) plays a fundamental role for binding to platelet GpIbα [35]. In WT-VWF, complex formation with GpIbα causes the α1-β2 loop to rotate away from GpIbα and shift by over 6 Å [35]. In unliganded A1, the loop sterically clashes with GpIbα, blocking its binding to the platelet receptor. The p.R1205X variants, and especially p.R1205L and p.R1205C, disrupt this motion by rotating the conformation of the α1-β2 loop (Fig 12, p.R1205L), thus preventing key interactions with K1332/R1334 and E225/D235 of GpIbα. Regarding the interaction of p.R1205C/L with platelet GPIb, the EAHAD Coagulation Factor Variant Database lacks specific data on potential impaired binding to GPIb and currently classifies these variants merely as “likely pathogenic”.

A pertinent question is why R1205 variants, situated in the D3 domain, predominantly affect the function of the A1-A2-A3 region rather than other domains like D4. The D4 domain, along with the C domains, is primarily responsible for propeptide interaction and multimer storage, functions distinct from the clearance and platelet adhesion mechanisms central to this study. Experimental evidence indicates that the A1-A2-A3 domains harbor the principal binding sites for macrophage receptors like LRP1, and the adjacent D’D3 domain serves a critical regulatory role. The R1205 residue acts as a structural hinge within this D’D3 regulatory unit; its mutation allosterically disrupts the restraint on the A1-A2-A3 domains, thereby enhancing LRP1 binding and impairing GPIbα interaction without directly involving the more C-terminal domains in the primary pathogenic mechanism.

In summary, our modeling investigation shows that R1205 in VWF’s D3 domain serves two critical functions: a) modulating LRP1 domain IV binding and thus the protein’s clearance and b) maintaining a conformational state competent for GPIbα engagement. These findings underscore how R1205-driven structural dynamics dictate VWF function, a paradigm disrupted by pathogenic variants at this position. These findings provide a basis for experimental validation to verify the predicted biochemical interactions and determine also the precise subtypes of these natural VWF variants and design therapeutics in these types of VWD.

## Materials and methods

### Molecular modeling pipeline

**Fig 13:**
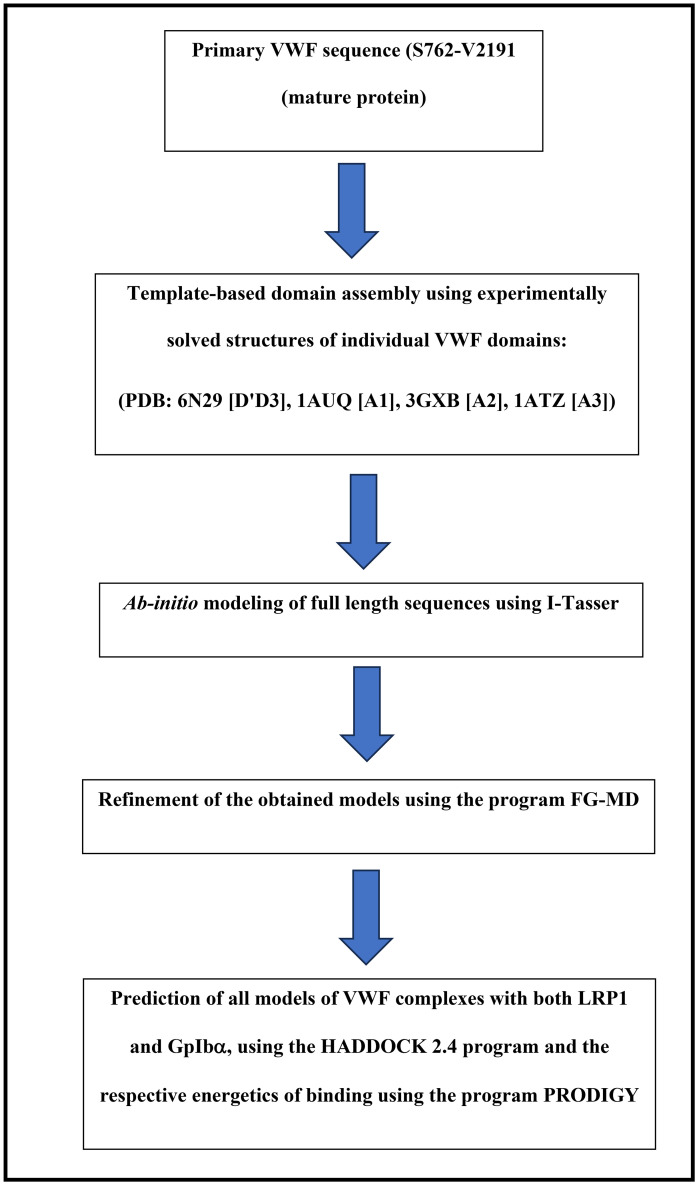
Flowchart of the entire pipeline of the modeling procedure.

Von Willebrand factor (VWF) is a large, multi-domain glycoprotein. While high-resolution structures are available for several isolated globular domains (D’D3, A1, A2, A3), the full-length protein and the structural context linking these domains remain poorly characterized. This gap is particularly critical for understanding the long-range conformational effects of variants like those at R1205, which resides in the D’D3 domain but can allosterically influence distal regions involved in ligand binding. To address this, we employed an integrative computational pipeline to construct a model of a large VWF segment (residues S764–V2191, numbering of the mature molecule) that encompasses the D’D3 through A3 domains. This segment includes the known structured domains connected by flexible linker regions, the conformation of which is crucial for the protein function but cannot be inferred from existing partial structures alone. Our modeling strategy proceeded as follows: 1. Template-Based Domain Assembly: we used the experimentally solved structures of the individual VWF domains (PDB: 6N29 [D’D3], 1AUQ [A1], 3GXB [A2], 1ATZ [A3]) as foundational templates. The known structured regions within our target sequence (S764–V2191) were initially aligned to these templates; 2. *ab initio* modeling of full-length segments: to predict the structure of the full segment, including the flexible linkers and the spatial arrangement of the globular domains relative to one another, we utilized the I-TASSER server. I-TASSER performs iterative threading and assembly refinement, effectively “linking” the known domain structures by predicting the conformation of the intervening sequences, thus generating a complete model; 3. model refinement: the initial I-TASSER models were subsequently refined using the FG-MD (Fragment-Guided Molecular Dynamics) program. FG-MD improves local geometry by identifying analogous structural fragments from the PDB and using them as spatial restraints to guide molecular dynamics simulations, thereby removing steric clashes and optimizing hydrogen-bonding networks; 4. validation of model quality: the quality of the final models for both wild-type (WT) and variant VWF was assessed using the Template Modeling Score (TM-score), a metric that evaluates the global topological similarity to native-like structures. A TM-score >0.5 indicates a model of correct topology; 5. protein-protein docking: the refined models of VWF species and the predicted structure of LRP1 Cluster IV were used as input for protein-protein docking using HADDOCK2.4. The latter is particularly suited for this task as it can integrate biochemical data and drive the docking process via ambiguous interaction restraints, which is ideal for systems where specific binding sites are not fully defined; 6. binding affinity prediction: finally, the binding affinity (ΔG) for the resulting complexes was predicted from their 3D coordinates using the PRODIGY server, which calculates interaction energies based on inter-residue contacts and physicochemical properties (see Fig 13 for the entire modeling pipeline). This structured approach allowed us to move beyond the limitations of isolated domain structures and generate a cohesive model capable of providing insights into the long-range structural and functional consequences of the R1205X variants.

### In-silico modeling of the missense VWF monomers and domain IV of LRP1.

Arginine-1205 (R1205) is localized in the D3 domain of VWF, whose X-ray structure was solved and deposited in the PDB website along with other domains of the mature protein: D’D3 (S764-P1241, PDB file “6N29”) [36], A1 (D1261-1468, PDB file “1AUQ”), A2 (M1495-H1674, PDB file “3GXB”) and A3 domains (D1685-S1873, PDB file “1ATZ”). Thus, we used the above X-ray-solved structures as templates, performing a molecular modeling of the stretched remaining sequences of the entire VWF region S1-V1428 (S764-V2191 for the full-length numbering system). Much progress has been made in protein structure prediction because of decades of effort [37–43]. Hence, the analysis of WT and pathological variants of VWF at R1205 was carried out using the I-TASSER threading modeling server (Iterative ThreadingASSEmbly Refinement) (http://zhanglab.ccmb.med.umich.edu/I-TASSER/), as previously detailed [44,45]. The program generated multiple models, all exhibiting an elongated VWF conformation that was consistent across species. This elongation broadly matched both the extended cryo-EM structures of VWF tubules and the shear force-induced uneven protomer extension observed in atomic force microscopy studies (see S8 Fig for partial comparison the experimental cryo-EM D’D3 structure and that of the entire D’D3-A1-A2-A3-D4 domain obtained in silico with I-TASSER [46,47]). Subsequently, each model was refined using the FG-MD program [48]. This program first identifies analogous fragments from the PDB by the structural alignment program TM-align [17]. Spatial restraints extracted from the fragments are then used to re-shape the funnel of the MD energy landscape and guide the molecular dynamics conformational sampling. Each molecular model of any VWF species was chosen only if TM-score was ≥ 0.5. This parameter is a proposed scale for measuring the structural similarity between two structures (in this case the predicted and the native structure of the solved VWF domains) [49]. Because the Root Mean Square Deviation (RMSD) is an average distance of all residue pairs in two structures, a local error (e.g., a misorientation of the tail) will raise a big RMSD value although the global topology is correct. In TM-score, however, the small distance is weighed stronger than the big distance which makes the score insensitive to the local modeling error. Hence, a TM-score ≥0.5 indicates a model of correct topology and a TM-score<0.17 means a random similarity. It has to be remarked that these cutoff values does not depends on the protein length [49]. FG-MD program aims at refining the initial models closer to the native structure. It can also improve the local geometry of the structures by removing the steric clashes and improving the torsion angle and the hydrogen-binding networks.

LRP1is a high molecular weight endocytic scavenger receptor that is highly expressed on cells of different tissues including macrophages [50]. The extracellular domain of LRP1 is structured on a modular structure consisting of four clusters (I, II, III and IV) of LDL receptor type A repeats that drive the interaction with several ligands [51]. In vitro studies have shown that cluster IV of LRP1 can bind to VWF [7,52] and that LRP1 modulates VWF endocytosis into early endosomes [51]. The binding of wild type VWF to LRP1 occurs only when the former is in the stretched conformation, induced by high shear stress or ristocetin [29,51]. No validated X-ray diffraction, NMR-based and cryo-EM structure of LRP1 is present in the PDB database. Hence, the extracellular CCR IV (Cluster IV of complement-like receptor) domain (cluster IV) of LRP1 (residues V3331-T3779; for details see S4 Table in the Supporting Information file) was modeled and refined by using the I-TASSER and FG-MD programs, as described above for the VWF monomer species.

### Molecular modeling of interaction between the VWF species and both LRP1 and GpIbα

Once both the VWF species and LRP1 Cluster IV models were refined, their docking complexes was investigated *in-silico* using the High Ambiguity Driven protein-protein DOCKing (HADDOCK) program version: 2.4 (https://rascar.science.uu.nl/HADDOCK2.4/) [53,54].

Docking was guided by ambiguous interaction restraints (AIRs) derived from known binding regions. The docking protocol involved rigid-body docking, semi-flexible refinement in torsion angle space, and final refinement in explicit solvent. Clusters were ranked based on the HADDOCK score, calculated as:


$$\text{HADDOCK}\;\text{score}=\text{1}.\text{0}\times {\text{E}}_{\text{vdw}}+\text{0}.\text{2}\times {\text{E}}_{\text{elec}}+\text{1}.\text{0}\times {\text{E}}_{\text{desol}}+\text{0}.\text{1}\times {\text{E}}_{\text{air}}$$

The top-ranking cluster (lowest HADDOCK score) and the highest number of observed clusters or populations were selected for further analysis [55].

For interactions between VWF species and the N-terminal domain of platelet GpIbα, the crystal structure of GpIbα (PDB: 1P9A) was used. Docking was performed similarly, and interaction interfaces were analyzed using PDBsum Generate (http://www.ebi.ac.uk/thornton-srv/databases/cgi-bin/pdbsum/GetPage.pl?pdbcode=index.html). The graphical rendering of the various models was performed using the PyMol program.

### Energetics of WT and VWF variants models with both LRP1 Cluster IV and N-terminal domain of GpIbα

Binding affinities (ΔG) for the complexes were predicted using PRODIGY, which calculates interaction energies based on inter-residue contacts and physicochemical properties. The PRODIGY algorithm uses structural features such as interface area and non-covalent interactions to estimate binding free energies [56]. All calculations were performed at 37°C.

## Supporting information

S1 Fig Magnification of the molecular model of the p.R1205H VWF variant (VWF “Vicenza”) showing the polar interaction of the side chain of H1205 with CO group of L1186. The model was obtained with the I-Tasser program, whereas the manipulation was accomplished with the Pymol software.
(DOCX)

S2 Fig Magnification of the molecular model of the p.R1205C VWF varant showing the polar interaction of the side chain of C1205 with E1200. The model was obtained with the I-Tasser program, whereas the manipulation was accomplished with the Pymol software.
(DOCX)

S3 Fig Magnification of the molecular model of the p.R1205L VWF varant showing that no interaction is present between the L1205 side chain and the surrounding aminoacids. The model was obtained with the I-Tasser program, whereas the manipulation was accomplished with the Pymol software.
(DOCX)

S4 Fig Magnification of the molecular models of the p.R1205S VWF variant showing the polar interactions (dashed lines) of the side chain of S1205 with S1208, and V1201. The models was obtained with the I-Tasser program, whereas the manipulation was accomplished with the Pymol software.
(DOCX)

S5 Fig Superposition of p.R1205H, p.R1205C, p.R1205L, and p.R1205S models with that of WT-VWF(764-2191). The side chain of R1205 is shown in all cases as yellow sticks, while the side chain of mutated amino acids is shown as red sticks. The superposition was accomplished with the Pymol program.
(DOCX)

S6 Fig Molecular model of the Domain IV of LRP1 (sequence V3331-P3880) obtained using I-Tasser platform The side chains of V3331 and P3780 are shown as red sticks. The rendering was accomplished with the Pymol program.
(DOCX)

S7 Fig A) Energetics of equilibrium interaction at 37 °C between WT and R1205 variant forms with the best in silico models of p.R1205H, p.R1205C, p.R1205L, and p.R1205S obtained with Haddock 2.4 program. B) Interface area of the various adducts between the VWF constructs and LRP1, calculated by the PDBSum program. (DOCX)

S8 Fig Partial superposition of the VWF D’D3 domain structure solved by cryo-EM (PDB 7WPQ, shown in cyan; the D1-D2 region was eliminated) and the model of the entire region D’D3-A1-A2-A3-D4 obtained by molecular modeling by I-TASSER (shown in green). The rendering was attained with the PyMol program.
(DOCX)

S1 Table PDB files for all obtained models of the wild-type (WT) and VWF variants, both as singular molecules and in complex with LRP1 domain IV and platelet GpIbα. (DOCX)

S2 Table Interface of VWF forms with LRP1. (DOCX)

S3 Table Statistics of docking results obtained with the HADDOCK program. (DOCX)

S4 Table Primary sequence (aa 3331-3780) of CCR IV of LRP1 used to model its 3D structure with I-TASSER. (DOCX)
