# Supplementary material for: Natural variants of von Willebrand factor R1205 causing von Willebrand disease with accelerated von Willebrand factor clearance: In silico docking models and energetics of the interaction with both LRP1 and GpIb A1 domain
Source: PLoS Comput Biol. 2025 Dec 3;21(12):e1013458. doi: 10.1371/journal.pcbi.1013458 (PMC12711066; doi:10.1371/journal.pcbi.1013458)
Supplement: S1 Fig — The model was obtained with the I-Tasser program, whereas the manipulation was accomplished with the Pymol software. (DOCX) [file pcbi.1013458.s001.docx]

**S1 Figure .** Magnification of the molecular model of the p.R1205H VWF variant (VWF “Vicenza”) showing the polar interaction of the side chain of H1205 with CO group of L1186. The model was obtained with the I-Tasser program, whereas the manipulation was accomplished with the Pymol software.
