# Supplementary material for: Natural variants of von Willebrand factor R1205 causing von Willebrand disease with accelerated von Willebrand factor clearance: In silico docking models and energetics of the interaction with both LRP1 and GpIb A1 domain
Source: PLoS Comput Biol. 2025 Dec 3;21(12):e1013458. doi: 10.1371/journal.pcbi.1013458 (PMC12711066; doi:10.1371/journal.pcbi.1013458)
Supplement: S2 Fig — The model was obtained with the I-Tasser program, whereas the manipulation was accomplished with the Pymol software. (DOCX) [file pcbi.1013458.s002.docx]

**S2 Figure.** Magnification of the molecular model of the p.R1205C VWF varant showing the polar interaction of the side chain of C1205 with E1200. The model was obtained with the I-Tasser program, whereas the manipulation was accomplished with the Pymol software.
