# Supplementary material for: Natural variants of von Willebrand factor R1205 causing von Willebrand disease with accelerated von Willebrand factor clearance: In silico docking models and energetics of the interaction with both LRP1 and GpIb A1 domain
Source: PLoS Comput Biol. 2025 Dec 3;21(12):e1013458. doi: 10.1371/journal.pcbi.1013458 (PMC12711066; doi:10.1371/journal.pcbi.1013458)
Supplement: S4 Fig — The models was obtained with the I-Tasser program, whereas the manipulation was accomplished with the Pymol software. (DOCX) [file pcbi.1013458.s004.docx]

**S4 Figure.** Magnification of the molecular models of the p.R1205S VWF variant showing the polar interactions (dashed lines) of the side chain of S1205 with S1208, and V1201. The models was obtained with the I-Tasser program, whereas the manipulation was accomplished with the Pymol software.
