# Supplementary material for: Natural variants of von Willebrand factor R1205 causing von Willebrand disease with accelerated von Willebrand factor clearance: In silico docking models and energetics of the interaction with both LRP1 and GpIb A1 domain
Source: PLoS Comput Biol. 2025 Dec 3;21(12):e1013458. doi: 10.1371/journal.pcbi.1013458 (PMC12711066; doi:10.1371/journal.pcbi.1013458)
Supplement: S5 Fig — The side chain of R1205 is shown in all cases as yellow sticks, while the side chain of mutated amino acids is shown as red sticks. The superposition was accomplished with the Pymol program. (DOCX) [file pcbi.1013458.s005.docx]

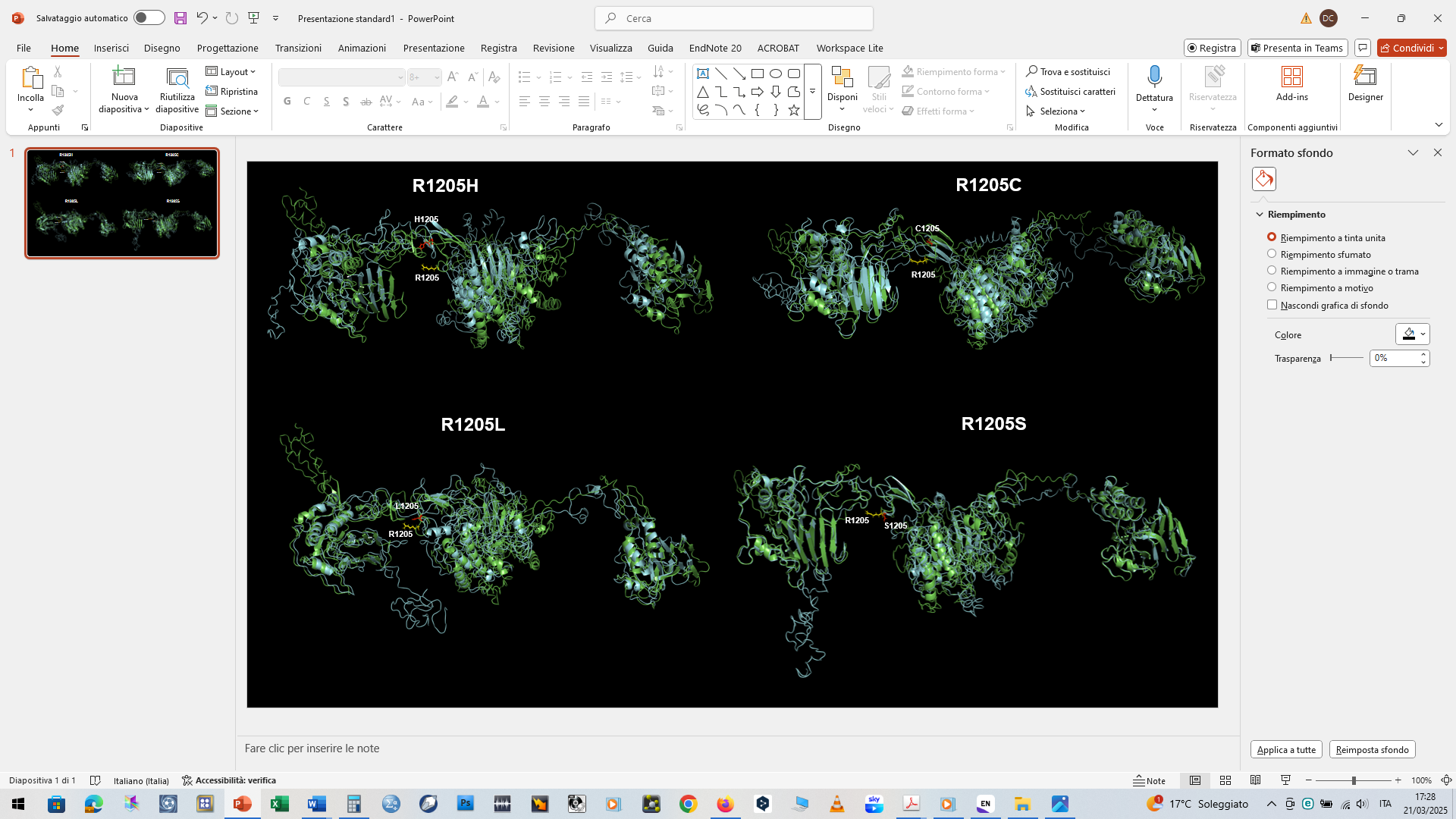


**S5 Figure**. Superposition of p.R1205H, p.R1205C, p.R1205L, and p.R1205S models with that of WT-VWF(764-2191). The side chain of R1205 is shown in all cases as yellow sticks, while the side chain of mutated amino acids is shown as red sticks. The superposition was accomplished with the Pymol program.
