# Supplementary material for: Natural variants of von Willebrand factor R1205 causing von Willebrand disease with accelerated von Willebrand factor clearance: In silico docking models and energetics of the interaction with both LRP1 and GpIb A1 domain
Source: PLoS Comput Biol. 2025 Dec 3;21(12):e1013458. doi: 10.1371/journal.pcbi.1013458 (PMC12711066; doi:10.1371/journal.pcbi.1013458)
Supplement: S6 Fig — The rendering was accomplished with the Pymol program. (DOCX) [file pcbi.1013458.s006.docx]

**S6 Figure**. Molecular model of the Domain IV of LRP1 (sequence V3331-P3880) obtained using I-Tasser platform The side chains of V3331 and P3780 are shown as red sticks. The rendering was accomplished with the Pymol program.
