# Supplementary material for: Natural variants of von Willebrand factor R1205 causing von Willebrand disease with accelerated von Willebrand factor clearance: In silico docking models and energetics of the interaction with both LRP1 and GpIb A1 domain
Source: PLoS Comput Biol. 2025 Dec 3;21(12):e1013458. doi: 10.1371/journal.pcbi.1013458 (PMC12711066; doi:10.1371/journal.pcbi.1013458)
Supplement: S7 Fig — (DOCX) [file pcbi.1013458.s007.docx]

**S7 Figure.** **A)** Energetics of equilibrium interaction at 37 °C between WT and R1205 variant forms with the best in silico models of p.R1205H, p.R1205C, p.R1205L, and p.R1205S obtained with Haddock 2.4 program. **B)** Interface area of the various adducts between the VWF constructs and LRP1, calculated by the PDBSum program.
