# Supplementary material for: Natural variants of von Willebrand factor R1205 causing von Willebrand disease with accelerated von Willebrand factor clearance: In silico docking models and energetics of the interaction with both LRP1 and GpIb A1 domain
Source: PLoS Comput Biol. 2025 Dec 3;21(12):e1013458. doi: 10.1371/journal.pcbi.1013458 (PMC12711066; doi:10.1371/journal.pcbi.1013458)
Supplement: S8 Fig — The rendering was attained with the PyMol program. (DOCX) [file pcbi.1013458.s008.docx]

**S8 Figure.** Partial superposition of the VWF D’D3 domain structure solved by cryo-EM (PDB 7WPQ, shown in cyan; the D1-D2 region was eliminated) and the model of the entire region D’D3-A1-A2-A3-D4 obtained by molecular modeling by I-TASSER (shown in green). The rendering was attained with the PyMol program.
