# Supplementary material for: Natural variants of von Willebrand factor R1205 causing von Willebrand disease with accelerated von Willebrand factor clearance: In silico docking models and energetics of the interaction with both LRP1 and GpIb A1 domain
Source: PLoS Comput Biol. 2025 Dec 3;21(12):e1013458. doi: 10.1371/journal.pcbi.1013458 (PMC12711066; doi:10.1371/journal.pcbi.1013458)
Supplement: S1 Table — (DOCX) [file pcbi.1013458.s009.docx]

**S1 Table**

**PDB files for all obtained models of the wild-type (WT) and VWF variants, both as singular molecules and in complex with LRP1 domain IV and platelet GpIbα. All the models may be downloaded from the *PDBsum* server at the following address:**

<https://www.ebi.ac.uk/thornton-srv/databases/pdbsum/Generate.html>

1. **I-TASSER models of single molecules**
2. **WT-VWF (764-2191):**

**PDB code: oiu4
Password: 073654**

1. **p. R1205H (764-2191):**

**PDB code: ojj4
Password: 155257**

1. **p.R1205C (764-2191):**

**PDB code: ojk1
Password: 165314**

1. **p.R1205L (764-2191):**

**PDB code: ojk2
Password: 165417**

1. **p.R120S (764-2191):**

**PDB code: ojl1
Password: 165331**

1. **Domain IV-LRP1 (V331–P3780)**

**PDB code: ojn1
Password: 182301**

1. **Haddock models of the VWF (WT- and variants)-domain IV of LRP1 adducts**
2. **WT-VWF-LRP1:**

**PDB code: ojl6
Password: 160841**

1. **R1205H-VWF-LRP1:**

**PDB code: ojl7
Password: 163353**

1. **R1205C-VWF-LRP1:**

**PDB code: ojl8
Password: 172117**

1. **R1205L-VWF-LRP1:**

**PDB code: ojl9
Password: 170433**

1. **R1205S-VWF-LRP1:**

**PDB code: ojm3
Password: 173745**

1. **Haddock models of the VWF (WT- and variant)-GpIbα adducts**
2. **WT-VWF-GpIb:**

**PDB code: ojn2
Password: 180710**

1. **p.R1205H-VWF-GpIb:**

**PDB code: ojn3
Password: 184824**

1. **p.R1205C-VWF-GpIb:**

**PDB code: ojn5
Password: 185135**

1. **p.R1205L-VWF-GpIb:**

**PDB code: ojn6
Password: 184644**

1. **p.R1205S-VWF-GpIb:**

**PDB code: ojn8
Password: 183655**
