# Supplementary material for: Natural variants of von Willebrand factor R1205 causing von Willebrand disease with accelerated von Willebrand factor clearance: In silico docking models and energetics of the interaction with both LRP1 and GpIb A1 domain
Source: PLoS Comput Biol. 2025 Dec 3;21(12):e1013458. doi: 10.1371/journal.pcbi.1013458 (PMC12711066; doi:10.1371/journal.pcbi.1013458)
Supplement: S2 Table — (DOCX) [file pcbi.1013458.s010.docx]

| \| Hydrogen bonds \| \| --- \| \| \| ## \| WT-VWF res. \| Dist. (Å) \| LRP1 res. \| \| --- \| --- \| --- \| --- \| \| 1 \| A:ARG 1392[ HE ] \| 1.74 \| B:ASP 435[OD1] \| \| 2 \| A:ARG 1392[HH12] \| 1.72 \| B:GLU 443[OE2] \| \| 3 \| A:ARG 1392[HH11] \| 2.49 \| B:GLU 444[OE2] \| \| 4 \| A:ARG 1392[HH21] \| 1.61 \| B:GLU 444[OE2] \| \| 5 \| A:ARG 1392[HH22] \| 2.25 \| B:ASP 435[OD1] \| \| 6 \| A:ARG 1395[HH12] \| 1.65 \| B:GLU 443[OE1] \| \| 7 \| A:ARG 1399[HH12] \| 1.57 \| B:ASP 442[OD2] \| \| 8 \| A:ARG 1399[HH11] \| 1.63 \| B:ASP 436[O] \| \| 9 \| A:ARG 1399[HH22] \| 2.22 \| B:ASP 442[OD1] \| \| 10 \| A:ARG1943[HE] \| 1.65 \| B:ASP 272[OD2] \| \| 11 \| A:ARG1943[HH21] \| 1.95 \| B:ASP 272[OD2] \| \|  \| Salt bridges \| \| \| \| \| --- \| --- \| --- \| --- \| \| ## \| WT-VWF res. \| Dist. (Å) \| LRP1 res. \| \| 1 \| A:LYS 1332[NZ] \| 3.62 \| B:ASP 3717[OD1] \| \| 2 \| A:ARG 1392[NE] \| 3.85 \| B:GLU 3773[OE2] \| \| 3 \| A:ARG 1392[NE] \| 2.69 \| B:ASP 3765[OD1] \| \| 4 \| A:ARG 1392[NH1] \| 2.75 \| B:GLU 3773[OE2] \| \| 5 \| A:ARG 1392[NH1] \| 3.25 \| B:GLU 3774[OE2] \| \| 6 \| A:ARG 1392[NH2] \| 2.64 \| B:GLU 3774[OE2] \| \| 7 \| A:ARG 1392[NH2] \| 3.07 \| B:ASP 3765[OD1] \| \| 8 \| A:ARG 1392[NE] \| 3.68 \| B:GLU 3763[OE1] \| \| 9 \| A:ARG 1392[NH1] \| 2.59 \| B:GLU 3763[OE1] \| \| 10 \| A:ARG 1399[NH1] \| 3.78 \| B:ASP 3772[OD1] \| \| 11 \| A:ARG 1399[NH1] \| 2.62 \| B:ASP 3772[OD2] \| \| 12 \| A:ARG 1399[NH2] \| 3.14 \| B:ASP 3772[OD1] \| \| 13 \| A:ARG 1399[NH2] \| 3.34 \| B:ASP 3772[OD2] \| \| 14 \| A:ARG1943 [NE] \| 2.57 \| B:ASP 3602[OD2] \| \| 15 \| A:ARG1943 [NH2] \| 2.80 \| B:ASP 3602[OD2] \|   **S2 Table**  **Interface of VWF forms with LRP1**   1. **WT-VWF** |  |
| --- | --- | --- | --- | --- | --- | --- | --- | --- | --- | --- | --- | --- | --- | --- | --- | --- | --- | --- | --- | --- | --- | --- | --- | --- | --- | --- | --- | --- | --- | --- | --- | --- | --- | --- | --- | --- | --- | --- | --- | --- | --- | --- | --- | --- | --- | --- | --- | --- | --- | --- | --- | --- | --- | --- | --- | --- | --- | --- | --- | --- | --- | --- | --- | --- | --- | --- | --- | --- | --- | --- | --- | --- | --- | --- | --- | --- | --- | --- | --- | --- | --- | --- | --- | --- | --- | --- | --- | --- | --- | --- | --- | --- | --- | --- | --- | --- | --- | --- | --- | --- | --- | --- | --- | --- | --- | --- | --- | --- | --- | --- | --- | --- | --- | --- | --- | --- | --- | --- | --- |

**Table S2 (cont.)**

1. **p.R1205H VWF**

| \| Hydrogen bonds \| \| --- \| \| \| ## \| p.R1205H-VWF res. \| Dist. (Å) \| LRP1 res. \|  \| \| --- \| --- \| --- \| --- \| --- \| \| 1 \| A:LYS 1407[HZ2] \| 1.60 \| B:GLU 3773[OE1] \|  \| \| 2 \| A:ILE 1509[N] \| 3.33 \| B:GLU 3774[OE1] \|  \| \| 3 \| A:LYS 1508[HZ1] \| 2.49 \| B:GLU 3774[OE2] \|  \| \| 4 \| A:ARG 1399[HE] \| 1.66 \| B:ASP 3775[OD1] \|  \| \| 5 \| A:ARG 1399[HH22] \| 2.08 \| B:ASP 3775[OD1] \|  \| \| 6 \| A:PHE1867[O] \| 2.06 \| B:LYS 3742[HZ1] \|  \| \| |
| --- | --- | --- | --- | --- | --- | --- | --- | --- | --- | --- | --- | --- | --- | --- | --- | --- | --- | --- | --- | --- | --- | --- | --- | --- | --- | --- | --- | --- | --- | --- | --- | --- | --- | --- | --- | --- | --- |

| Salt bridges |
| --- |
| \| ## \| p.R1205H-VWF res. \| Dist. (Å) \| LRP1 res. \| \| --- \| --- \| --- \| --- \| \| 1 \| A:LYS 1407[NZ] \| 2.64 \| B:GLU 3773[OE1] \| \| 2 \| A:LYS 1407[NZ] \| 2.93 \| B:GLU 3773[OE2] \| \| 3 \| A:LYS 1508[NZ] \| 3.45 \| B:GLU 3773[OE2] \| \| 4 \| A:LYS 1508[NZ] \| 3.32 \| B:GLU 3774[OE2] \| \| 5 \| A:ARG 1399[NE] \| 2.61 \| B:ASP 3775[OD1] \| \| 6 \| A:ARG 1399[NH2] \| 2.92 \| B:ASP 3775[OD1] \| \| 7 \| A:ARG 1399[NE] \| 3.28 \| B:ASP 3775[OD2] \| |
|  |

| Hydrogen bonds | | | |
| --- | --- | --- | --- |
| ## | p.R1205C-VWF res. | Dist. (Å) | LRP1 res. |
| 1 | A:LYS 1408[HZ2] | 1.58 | B:ASP 3515[OD2] |
| 2 | A:ARG 1426[HH22] | 2.32 | B:GLY 3516[O] |
| 3 | A:ARG 1426[HH21] | 2.37 | B:GLU 3526[OE1] |
| 4 | A:LYS1913[HZ1] | 2.49 | B:CYS 3494[O] |
| 5 | A:LEU 1460[O] | 2.05 | B:ARG 3591[HH11] |
| 6 | A:GLN 1475[O] | 3.47 | B:THR 3535[OG1] |
| 7 | A:THR1901[O] | 2.14 | B:ARG 3469[HE] |
| 8 | A:GLN1909[OE1] | 3.73 | B:CYS 3507[SG] |

1. **p.R1205C VWF**

| Salt bridges | | | |
| --- | --- | --- | --- |
| ## | p.R1205C-VWF res. | Dist. (Å) | LRP1 res. |
| 1 | A:LYS 1408[NZ] | 2.58 | B:ASP 3515[OD2] |
| 2 | A:ARG 1426[NH2] | 2.79 | B:GLU 3526[OE1] |

**Table S2 (cont.)**

1. **p.R1205L VWF**

| ## | p.R1205L-VWF res. | Dist. (Å) | | LRP1 res. |
| --- | --- | --- | --- | --- |
| 1 | A:GLN 1168[N] | 2.76 | B:ASP  3354[O] | |
| 2 | A:GLN 1168[N] | 3.87 | B:THR  3355[O] | |
| 3 | A:ARG 1308[HH11] | 1.65 | B:ASP 3772[OD1] | |
| 4 | A:ARG 1308[HH21] | 1.56 | B:ASP 3772[OD2] | |
| 5 | A:ARG 1308[HH22] | 2.42 | B:GLU 3773[OE1] | |
| 6 | A:ARG 1308[HE] | 1.65 | B:GLU 3773[OE1] | |
| 7 | A:ARG 1399[HH11] | 1.74 | B:GLU 3773[OE2] | |
| 8 | A:ARG 1399[HE] | 1.85 | B:GLU 3774[OE2] | |
| 9 | A:ARG 1399[HH22] | 2.07 | B:GLU 3774[OE2] | |
| 10 | A:GLU 954[OE2] | 1.93 | B:LYS 3352[HZ2] | |

Hydrogen bonds

1. **p.R1205S VWF**

Hydrogen bonds

| \|  \| \| --- \| \| \| ## \| p.R1205S-VWF res. \| Dist. (Å) \| LRP1 res. \| \| --- \| --- \| --- \| --- \| \| 1 \| A:ARG1943[HE] \| 2.42 \| B:ASN  3397[O] \| \| 2 \| A:ARG1943[HH21] \| 2.28 \| B:CYS  3399[O] \| \| 3 \| A:ARG 1399[HH12] \| 1.67 \| B:ASN 3456[O] \| \| 4 \| A:SER 1345[OG] \| 3.30 \| B:CYS 3394[O] \| \| 5 \| A:TYR2082[HH] \| 1.90 \| B:THR 3739[O] \| \| 6 \| A:ASN 1396[OD1] \| 2.36 \| B:ARG3506[HH11] \| \| 7 \| A:GLU1981[OE2] \| 1.59 \| B:LYS3742[HZ3] \| \| |
| --- | --- | --- | --- | --- | --- | --- | --- | --- | --- | --- | --- | --- | --- | --- | --- | --- | --- | --- | --- | --- | --- | --- | --- | --- | --- | --- | --- | --- | --- | --- | --- | --- | --- | --- |

| Salt bridges |
| --- |
| \| ## \| p.R1205S-VWF res. \| Dist. (Å) \| LRP1 res. \| \| --- \| --- \| --- \| --- \| \| 1 \| A:GLU1981[OE2] \| 2.61 \| B:LYS 3742[NZ] \| |
