# Supplementary material for: Natural variants of von Willebrand factor R1205 causing von Willebrand disease with accelerated von Willebrand factor clearance: In silico docking models and energetics of the interaction with both LRP1 and GpIb A1 domain
Source: PLoS Comput Biol. 2025 Dec 3;21(12):e1013458. doi: 10.1371/journal.pcbi.1013458 (PMC12711066; doi:10.1371/journal.pcbi.1013458)
Supplement: S3 Table — (DOCX) [file pcbi.1013458.s011.docx]

**S3 Table. Statistics of docking results obtained with the HADDOCK program**

**S3 Table. Legend**: Cluster size refers to the number of distinct conformations or poses observed within the ensemble of generated protein-protein complexes [Mohammadi S, et al. Sci Rep. 2022;12(410):1-1534]. A larger cluster size indicates greater structural diversity, suggesting multiple binding modes or orientations between the proteins. RMSD values measure the variations or discrepancies in structure among distinct conformations within a given cluster. Reduced RMSD values indicate in Å limited deviation or greater structural similarity among various conformations, implying heightened stability and consistency in the binding interactions observed throughout the simulation period [Kufareva I, et al. Methods of Protein Structure Comparison. Methods in molecular biology (Clifton, NJ). 2012;857:231-57.].
