# Supplementary material for: Natural variants of von Willebrand factor R1205 causing von Willebrand disease with accelerated von Willebrand factor clearance: In silico docking models and energetics of the interaction with both LRP1 and GpIb A1 domain
Source: PLoS Comput Biol. 2025 Dec 3;21(12):e1013458. doi: 10.1371/journal.pcbi.1013458 (PMC12711066; doi:10.1371/journal.pcbi.1013458)
Supplement: S4 Table — (DOCX) [file pcbi.1013458.s012.docx]

| S4 Table. Primary sequence (aa 3331-3780) of CCR IV of LRP1 used to model its 3D structure with I-TASSER |
| --- |
| VSNCTASQFVCKNDKCIPFWWKCDTEDDCGDHSDEPPDCPEFKCRPGQFQCSTGICTNPAFICDGDNDCQDNSDEANCDIHVCLPSQFKCTNTNRCIPGIFRCNGQDNCGDGEDERDCPEVTCAPNQFQCSITKRCIPRVWVCDRDNDCVDGSDEPANCTQMTCGVDEFRCKDSGRCIPARWKCDGEDDCGDGSDEPKEECDERTCEPYQFRCKNNRCVPGRWQCDYDNDCGDNSDEESCTPRPCSESEFSCANGRCIAGRWKCDGDHDCADGSDEKDCTPRCDMDQFQCKSGHCIPLRWRCDADADCMDGSDEEACGTGVRTCPLDEFQCNNTLCKPLAWKCDGEDDCGDNSDENPEECARFVCPPNRPFRCKNDRVCLWIGRQCDGTDNCGDGTDEEDCEPPTAHTTHCKDKKEFLCRNQRCLSSSLRCNMFDDCGDGSDEEDCSIDP |
